# Proposing a validation scheme for ^13^C metabolite tracer studies in high-resolution mass spectrometry

**DOI:** 10.1007/s00216-019-01773-7

**Published:** 2019-04-10

**Authors:** Michaela Schwaiger-Haber, Gerrit Hermann, Yasin El Abiead, Evelyn Rampler, Stefanie Wernisch, Kelli Sas, Subramaniam Pennathur, Gunda Koellensperger

**Affiliations:** 1 Department of Analytical Chemistry, Faculty of Chemistry, University of Vienna, Waehringer Str. 38, 1090 Vienna, Austria; 2 Division of Nephrology, Department of Internal Medicine, University of Michigan, 1000 Wall St., Ann Arbor, MI 48105 USA; 3 ISOtopic solutions, Waehringer Str. 38, 1090 Vienna, Austria; 4 Vienna Metabolomics Center (VIME), University of Vienna, Althanstraße 14, 1090 Vienna, Austria; 5 Chemistry Meets Microbiology, Althanstraße 14, 1090 Vienna, Austria; 6 Department of Molecular and Integrative Physiology, University of Michigan, 1000 Wall St, Ann Arbor, MI 48105 USA

**Keywords:** Metabolomics, Stable isotope tracer experiments, Stable isotope labeling experiments, Mass spectrometry, Carbon isotopologue distribution, ^13^C labeling

## Abstract

^13^C metabolite tracer and metabolic flux analyses require upfront experimental planning and validation tools. Here, we present a validation scheme including a comparison of different LC methods that allow for customization of analytical strategies for tracer studies with regard to the targeted metabolites. As the measurement of significant changes in labeling patterns depends on the spectral accuracy, we investigate this aspect comprehensively for high-resolution orbitrap mass spectrometry combined with reversed-phase chromatography, hydrophilic interaction liquid chromatography, or anion-exchange chromatography. Moreover, we propose a quality control protocol based on (1) a metabolite containing selenium to assess the instrument performance and on (2) in vivo synthesized isotopically enriched *Pichia pastoris* to validate the accuracy of carbon isotopologue distributions (CIDs), in this case considering each isotopologue of a targeted metabolite panel. Finally, validation involved a thorough assessment of procedural blanks and matrix interferences. We compared the analytical figures of merit regarding CID determination for over 40 metabolites between the three methods. Excellent precisions of less than 1% and trueness bias as small as 0.01–1% were found for the majority of compounds, whereas the CID determination of a small fraction was affected by contaminants. For most compounds, changes of labeling pattern as low as 1% could be measured.

**Electronic supplementary material:**

The online version of this article (10.1007/s00216-019-01773-7) contains supplementary material, which is available to authorized users.

**Figure:**
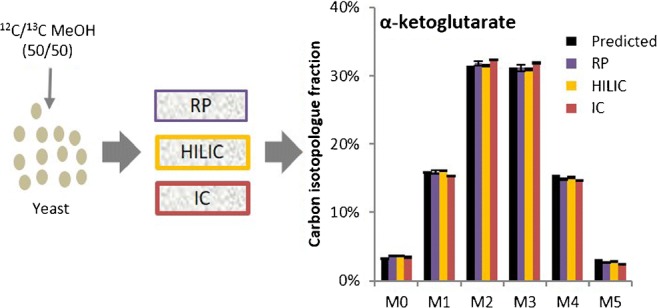
Graphical abstract

## Introduction

The metabolome is an inherently dynamic system, characterized by strongly regulated reaction rates, i.e., fluxes through a network of biochemical reactions [1]. Understanding regulation of these metabolic fluxes is an emerging topic in many key applications of metabolomics. Measuring metabolite concentration levels does not provide information about the intracellular dynamics [2, 3]. In fact, assessment of the intracellular flux configuration requires the application of more sophisticated approaches such as stable isotope labeling (tracer) experiments. The incorporation of labeled substrates into intermediates of the metabolic network introduces significantly altered isotopologue patterns, which enable resolution of fluxes—either on a qualitative or quantitative basis. In the former case, the dependence of pathways on different carbon sources and/or the partitioning of fluxes into diverging pathways at a branch point is inferred. More sophisticated quantitative flux evaluation can be achieved when the measurements of labeling patterns are combined with mathematical models of the network [4, 5]. The reliability of these approaches critically depends not only on the measurements as discussed later but also on a priori knowledge of the metabolic network and investigated conditions. This makes upfront planning of the experiments more challenging compared to other metabolomic applications. Efforts to provide heuristics for choosing a suitable tracer and for interpreting the resulting labeling patterns have facilitated isotope tracing experiments and will allow a broader community to take advantage of this approach [2]. The analysis of isotopologue distributions has also been incorporated into untargeted metabolomics software such as X13CMS [6], geoRge [7], Thermo Scientific’s Compound Discoverer 3.0, and Agilent Technologies’ MassHunter Profinder/Vista Flux. Although there are attempts to combine tracer studies with untargeted data analysis [6–8], the majority of labeling experiments are followed by targeted analysis. As comprehensively reviewed elsewhere [2, 9–11], the most commonly assessed metabolites in tracer experiments are organic acids, sugar (phosphates), nucleotides, and amino acids. One of the most frequently studied pathways is the tricarboxylic acid (TCA) cycle, frequently assessed via ^13^C-labeled glucose or glutamine. Those labeled tracers can be either fully or positionally enriched [3, 12]. The metabolite readouts include succinate, malate, citrate, and alpha-ketoglutarate [2]. Importantly, these small organic acids are frequently observed as unlabeled contaminants originating from outside the biological sample and have to be evaluated carefully [13].

The analytical task of measuring a significant, metabolite-specific alteration of the natural isotope pattern requires accurate determination of isotope ratios. Mass spectrometry is the established method in this field [12, 13]. Utilizing high-resolution mass spectrometry in full scan mode enables the determination of isotopologue distributions. In selected cases, additional fragmentation allows one to infer even positional, i.e., isotopomer, information [14, 15]. In this work, with reference to the metabolite panel that is key in different tracer studies (see Electronic Supplementary Material (ESM) Fig. S1) [2, 9–11], we address best practice guidelines for analytical method selection and method validation when planning such experiments.

Spectral accuracy is crucial when addressing tracer studies [16, 17]. Trueness and precision ultimately determine the minimum change of fractional isotopologue abundance that can be measured significantly. Factors to be considered include the instrument’s performance, the isotopologue pattern itself, the concentration of the molecule in question, and the procedural blanks and the sample itself, which might introduce an isotopologue-specific background.

A small common organic molecule containing the elements C, H, O, N, P, and S shows an isotopologue pattern with a high abundance first isotopologue (M0 containing only ^12^C, ^1^H, ^16^O, ^14^N, ^31^P, ^32^S) as all elements have no or comparably low abundance heavier isotopologues (named M1, M2, etc.). Hence, the dynamic range of the measurement becomes a limitation regarding both trueness and precision of assessed isotopologue distributions. In fact, for molecules in the nanomolar to low micromolar concentration range, heavy isotopologues are often below the limit of detection when using LC-MS [18]. On top of that, in high-resolution orbitrap measurements overestimation of the (most abundant) isotopologue has been discussed [16]. As a consequence, addressing analytical figures of merit of tracer experiments should involve standards with tailored isotopologue patterns different from the natural distribution. In pioneering studies, Portais and co-workers introduced in vivo synthesized metabolites with predictable specific patterns [13, 19]. Yeast or bacteria, commonly serving for the production of fully ^13^C-labeled internal standards [20], were utilized as small bioreactors to produce a biomass suitable for validation of tracer studies. Predictable specific labeling patterns were achieved upon fermentation on well-defined labeled substrate mixtures [13, 19]. In a seminal study, the group showed the potential of the validation approach focusing on amino acids [19]. While Portais and co-workers used *Pichia augusta* and *E. coli* [13, 19], Mairinger et al. implemented *Pichia pastoris* in GC-MS and LC-TOF-MS [21, 22].

In this work, we advance validation methods based on the in vivo synthesized ^13^C-labeled in-house reference material produced in *P. pastoris* and prove its validity on a panel of over 40 metabolites using three different LC separations combined with high-resolution orbitrap MS. Reversed-phase (RP) chromatography, hydrophilic interaction liquid chromatography (HILIC), and anion-exchange chromatography (IC) were investigated. These methods are discussed regarding their suitability in metabolomic studies utilizing stable isotopes. In addition to the sophisticated isotopologue-specific validation scheme, we propose the implementation of selenium-containing metabolites as quality control standards. Owing to the isotopic pattern of selenium, these compounds offer ideal reference patterns for carbon isotopologue distributions (CID) found in ^13^C tracer studies.

## Materials and methods

### Standards and solvents

Acetonitrile (ACN), methanol (MeOH), and water were of LC-MS grade and ordered from Fisher Scientific (Vienna, Austria) or Sigma Aldrich (Vienna, Austria). Ammonium bicarbonate, ammonium formate, and ammonium hydroxide were ordered as eluent additive for LC-MS from Sigma Aldrich. Formic acid was also of LC-MS grade and was ordered from VWR International (Vienna, Austria). For anion-exchange chromatography water from an ELGA water purification system was used (18.2 MΩ cm^−1^).

Natural abundance metabolite standards were purchased from Sigma Aldrich (Vienna, Austria) or Carbosynth (Berkshire, UK). They were weighed, dissolved in an appropriate solvent (water or 0.1 M hydrochloric acid for some amino acids), and combined to a multi-metabolite mix. Dilutions between 0.01 and 25 μM were prepared in either water (for RP and IC) or 50% ACN (for HILIC) dependent on the LC starting conditions. Selenomethionine (Se-Met) was prepared as a 10 μM single standard in water (for flow injection analysis and RP) and 50% ACN for HILIC.

### ^13^C-labeled in-house reference material

In collaboration with ISOtopic solutions e.U., the yeast *P. pastoris*, a methylotroph organism, was fermented on a mixture of natural abundance methanol and ^13^C methanol to produce the in-house reference material. ^1^H NMR measurements of the methanol mixture revealed a ratio of 50.245% (± 0.009%) ^12^C methanol and 49.755% (± 0.009%) ^13^C methanol. Those values were used to calculate the theoretical carbon isotopologue distribution (CID). A molecule with *n* carbon atoms will show an isotopologue distribution according to the *n*^th^ row of Pascal’s triangle [13]. The software @RISK (Palisade) was used to predict the error of the CIDs using Monte Carlo simulation with 1000 iterations. The model equation was ${M}_k=\left(\genfrac{}{}{0pt}{}{n}{k}\right)\ {p}^k{\left(1-p\right)}^{n-k}$ as described previously [13] with *p* = 0.49755. A dried aliquot of the extract of about one billion yeast cells (estimated by cell counting using a hemocytometer) was reconstituted in 200 μL water or 50% ACN (dependent on LC separation), centrifuged (4350×*g*, 4 °C, 15 min), and diluted 1:2 and 1:5 in the respective solvent.

### Preparation of mammalian cancer cell extract

In order to be able to compare the data with a mammalian cell extract, 5 × 10^5^ cells of the human colon cancer cell line HCT 116, acquired from ATCC (American Type Culture Collection, USA), were seeded in 6-well plates. After 24 h incubation, the cells were washed with phosphate buffered saline (PBS), quenched in liquid nitrogen, and extracted with 2 mL 80% cold MeOH. After centrifugation, 1 mL of the supernatant was dried and reconstituted in 200 μL water (for RP/IC) or 50% ACN (HILIC), respectively. Additionally, blank extractions of a 6-well plate were performed and processed in the same way as the cell extracts.

### Reversed-phase chromatography

Reversed-phase separation was performed with a Vanquish Horizon UPLC system on a fully wettable Acquity HSS T3 column (2.1 × 150 mm, 1.8 μm particle size, Waters) operated at a flow rate of 0.300 mL min^−1^ as previously published [23]. The column temperature was 40 °C and the injection volume 5 μL. Mobile phase A was 0.1% formic acid in water and B was 100% methanol (MeOH). Gradient elution started from 0 to 2.0 min with 0% B min; from 2.0 to 5.0 min ramp to 40% B, then increase to 100% at 5.8 min, until 7.8 min 100% B, 7.9 min decrease to 0% B followed by re-equilibration at 0% B for 4.1 min. This resulted in a total run time of 12 min. The injection volume was 5 μL and the injector needle was washed with ACN/MeOH/H_2_O 1:1:1 (v/v/v) for 5 s prior to each injection.

### Hydrophilic interaction liquid chromatography

A SeQuant® ZIC®-pHILIC column (150 × 2.1 mm, 5 μm, polymer, Merck-Millipore) was used with gradient elution under alkaline conditions. Mobile phase A was 90% 10 mM ammonium bicarbonate, pH 9.2/10% acetonitrile (ACN) and mobile phase B was 100% ACN. A Vanquish Horizon UPLC system was used at a flow rate of 0.300 mL min^−1^ and 40 °C. The following gradient was applied: 0.0–6.0 min 75–45% B, 6.0–7.0 min decrease to 30% B, 7.0–10.0 min 30% B, and at 10.0 min switch to 75% B and re-equilibration until 22 min. The injection volume was 5 μL and the injector needle was washed with ACN/MeOH/H_2_O 1:1:1 (v/v/v) for 5 s prior to each injection.

### Anion-exchange chromatography

The method was adapted from a previously published one [24]. A Dionex ICS 5000^+^ System (Thermo Scientific) was used for anion-exchange chromatography. The separation was conducted on a Dionex IonPac AS11-HC column (2 × 250 mm, 4 μm particle size, Thermo Scientific) equipped with a guard column, packed with the same material (2 × 50 mm), at 30 °C. A sodium hydroxide (NaOH) gradient was produced by a gradient pump using 10 mM and 100 mM NaOH. The separation was carried out at a flow rate of 0.380 mL min^−1^ beginning with 10 mM KOH over 3 min, 10–50 mM from 3 to 12 min, 50–100 mM from 12 to 19 min, held at 100 mM from 19 to 21 min, and re-equilibrated at 10 mM for 4 min. This resulted in a total run time of 25 min. A Dionex AERS 500, 2 mm suppressor was operated with 95 mA at a temperature of 15 °C. Methanol was provided as make-up flow at a flow rate of 0.150 mL min^−1^ from a Vanquish binary pump (Thermo Scientific) equipped with a restriction capillary (50 μm × 550 mm) to ensure stable backpressure. The samples were introduced via a Dionex AS-AP autosampler with water as transfer solvent/needle wash and partial loop injection of 5 μL.

### High-resolution mass spectrometry

High-resolution mass spectrometry was conducted on a high field Thermo Scientific™ Q Exactive HF™ quadrupole-Orbitrap mass spectrometer equipped with an electrospray source. Details on ESI source parameters for the three different LC separations are given in the ESM (Table S1). Full scan data were acquired in profile mode in the range of 65–750 *m/z* with resolutions of 120 and 30 K. The automatic gain control (AGC) target was set to 1e6. For RP, positive and negative mode data were acquired separately, whereas broader peaks in HILIC enabled fast polarity switching maintaining more than 15 data points per ionization mode across the chromatographic peak. For IC, only negative mode data were acquired since metabolites that would be better ionizable in positive mode are typically not detectable because of the suppressor.

### Data evaluation

All results are based on chromatographic peak areas received from targeted data evaluation in Tracefinder 4.1 (Thermo Scientific). Slight smoothing as implemented in peak detection by Tracefinder (5 points) was applied to improve automated peak integration. AccuCor, an open-source R-based algorithm, was used for natural isotope correction for high-resolution MS data taking into account whether isotopologues with the same nominal mass are resolved at a specific resolution [16]. For the in-house reference material used in this study, the carbon pattern was exactly predicted. Thus, no correction for the natural carbon isotope distribution was necessary. More precisely, applying a natural abundance correction for carbon would have introduced an error for this labeled reference material. Thus, the published R script was slightly adapted, i.e., the natural abundance of carbon was changed to 100% ^12^C, before running the AccuCor algorithm which also automatically calculated the CIDs.

## Results and discussion

### Measuring isotopologue distributions in high-resolution mass spectrometry

Uncertainty and accuracy are undoubtedly key metrics defining the relevance of changes of CIDs as encountered in ^13^C tracer studies. However, experimental evaluation of these metrics is not a straightforward task. When measuring the natural isotopic distribution of the two most abundant isotopologues of a common small organic molecule by state-of-the-art orbitrap technology, excellent precisions (typically 0.05% as observed in this study) can be obtained. For example, measuring a 10 μM standard solution of methionine (C_5_H_11_NO_2_S) delivered excellent precisions as low as 0.02% and a trueness bias of 0.5% considering the two most abundant isotopologues (*N* = 4). We obtained 95.3% of M0 and 4.7% of M1 compared to the theoretical values 94.8% and 5.2%, respectively. However, the CID determination considering all heavy isotopologues of a common organic molecule of natural abundance requires a linear dynamic measurement range of more than five orders of magnitude, which is beyond the capabilities of high-resolution mass spectrometry. For instance, measuring all isotopologues of a six-carbon molecule would require measuring over 10 orders of magnitude to assess both the highest and the lowest abundance isotopologue. Therefore, metabolite standards with natural CIDs are not ideal validation tools in tracer studies, where otherwise low abundance isotopologues are enriched.

**Fig. 1:**
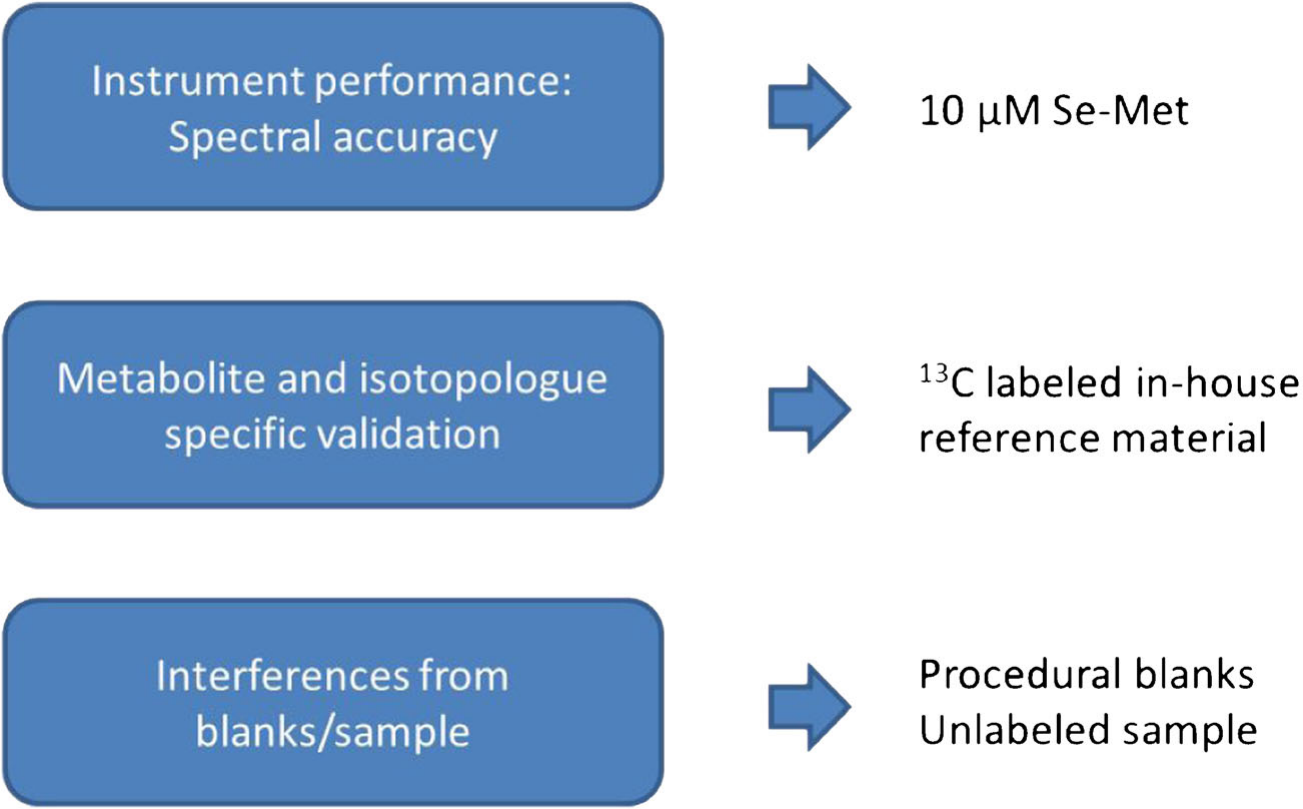
Proposed validation scheme addressing uncertainty and accuracy in ^13^C tracer studies. A selenomethionine (Se-Met) standard was used to evaluate the instrument performance regarding spectral accuracy. An in-house reference material provides metabolite- and isotopologue-specific method validation and procedural blanks/an unlabeled sample can reveal possible interferences from endogenous contamination

In this work, a tailored validation scheme is proposed addressing uncertainty and accuracy in ^13^C tracer studies (Fig. 1). In the first step, a standard was introduced to evaluate instrument performance regarding spectral accuracy using different chromatographic separations or without separation. Ideal case conditions, for such experiments, are micromolar concentrations of a compound with good ionization and a known isotopic pattern (not a carbon pattern) that is representative of biologically relevant isotopologue distributions, i.e., chemically similar regarding the number of isotopologues and ratios. As a second benchmark material, a labeled material containing all metabolites of interest was included for metabolite- and isotopologue-specific method validation (denoted as in-house reference material). Finally, the scheme included the measurement of procedural blanks and unlabeled samples to reveal possible interferences from endogenous contamination introducing a trueness bias. More specifically, to realize this validation scheme, we used selenomethionine to benchmark the mass spectrometer performance, a ^13^C-labeled in-house reference material produced in the yeast *P. pastoris* and a cell extract of HCT 116 human colon cancer cells together with extraction blanks.

### Addressing instrument performance regarding spectral accuracy

The selenium-containing organic molecule selenomethionine (Se-Met, C_5_H_11_NO_2_Se) constitutes an ideal compound for ESI-MS and can be measured in both positive and negative mode. Compared to the isotopic pattern of C, H, N, O, and P, selenium possesses a unique isotopic pattern featuring six isotopes of greater than 1% natural abundance [25]. These characteristics were exploited to assess the spectral accuracy of the mass spectrometer. The theoretical pattern of selenomethionine was calculated considering the resolution used for data acquisition with enviPat [26]. The six main isotopologues arising from the selenium isotopes ^74^Se, ^76^Se, ^77^Se, ^78^Se, ^80^Se, and ^82^Se were included and, considering ^12^C, ^1^H, ^14^N, ^16^O, their sum was set as 100%. Slight differences in the calculated pattern for resolutions of 120 and 30 K originate from insufficient resolution of other isotopologues than selenium, i.e., ^13^C, ^2^H, ^15^N, ^18^O (natural abundance correction for Se-containing metabolites was not implemented in AccuCor at the time of this study and hence could not be performed).

**Fig. 2:**
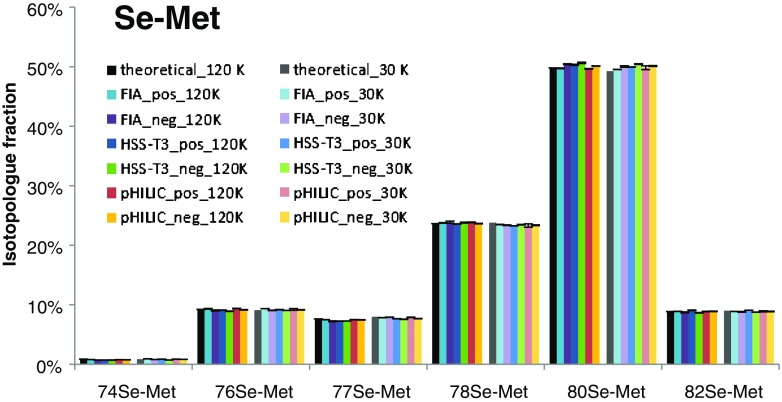
Isotopologue distribution of a 10 μM standard of selenomethionine (Se-Met) measured by flow injection analysis (FIA) as well as reversed-phase (HSS-T3) and HILIC (ZIC pHILIC) separation in both positive and negative electrospray ionization with resolutions of 120 and 30 K on a Q Exactive HF orbitrap mass spectrometer in full MS mode. Black: theoretical distribution of Se-Met considering the six highest abundance selenium isotopes. The trueness bias (difference between theoretical and measured ID) was less than 1% for all isotopes at both polarities and resolutions. The error bars denote the standard deviation of the technical replicates (*N* = 4) which was less than 0.2% for investigated isotopes

A 10 μM standard of Se-Met was measured with flow injection analysis (FIA) prior to any LC-MS measurements. Additionally, it was then also included as a single standard in the performed LC separations, i.e., RP, HILIC, and IC. Figure 2 shows the measured isotopologue distributions (IDs) in comparison to the theoretical one considering the six isotopic MS peaks resulting from the selenium pattern.

As can be readily observed (Table 1), the obtained accuracy was excellent with a trueness bias (difference between theoretical and measured ID) of less than 1% and a precision (standard deviation) of less than 0.2% (four technical replicates). This is true for positive and negative ESI as well as polarity switching, which was applied for the HILIC separation. The data show that Se-Met constitutes an ideal compound to evaluate the accuracy (including trueness bias and precision) of isotopologue distributions of a mass spectrometer. The standard could be implemented in all metabolomics workflows, besides anion-exchange chromatography. In the latter case, Se-Met was lost as a result of the suppression system as were all other amino acids. When this approach was used, spectral accuracy could only be addressed by preliminary FIA.

**Table 1:** Isotopologue distributions (ID) of selenomethionine measured by flow injection analysis (FIA) in positive and negative ESI mode with 120 and 30 K resolution on a Q Exactive HF orbitrap mass spectrometer (*N* = 4) (Meas. ID) with their trueness bias in comparison to the theoretical pattern (Theor. ID) of Se-Met and precision (Prec.)

Isotopologue	Theor. ID	Meas. ID	Trueness bias	Prec.	Meas. ID	Trueness bias	Prec.
	120 K	FIA pos 120 K			FIA neg 120 K		
^74^Se-Met	0.9	0.8	−0.1	0.01	0.7	−0.2	0.02
^76^Se-Met	9.2	9.3	0.1	0.06	9.0	−0.2	0.10
^77^Se-Met	7.6	7.5	−0.1	0.02	7.2	−0.4	0.07
^78^Se-Met	23.6	23.8	0.1^a^	0.05	23.9	0.2^a^	0.18
^80^Se-Met	49.8	49.7	−0.1	0.06	50.4	0.6	0.08
^82^Se-Met	8.9	8.9	0.0	0.02	8.7	−0.1^a^	0.09
	30 K	FIA pos 30 K			FIA neg 30 K		
^74^Se-Met	0.9	0.9	0.1^a^	0.01	0.8	−0.1	0.01
^76^Se-Met	9.1	9.4	0.3	0.01	9.1	0.0	0.03
^77^Se-Met	8.0	7.8	−0.2	0.02	7.9	−0.1	0.02
^78^Se-Met	23.8	23.5	−0.3	0.04	23.4	−0.4	0.05
^80^Se-Met	49.3	49.5	0.3^a^	0.03	50.0	0.8^a^	0.12
^82^Se-Met	9.0	8.9	−0.1	0.01	8.8	−0.2	0.06

Values are in percent (%) of the sum (100%) of all six isotopologues (for theoretical and measured ID). Trueness bias as difference between theoretical and measured value and precision as standard deviation of technical replicates are also given in percent (%). Trueness bias = Isotopologue fraction_meas._ − Isotopologue fraction_theor_

^a^Deviations between the difference of theoretical ID and measured ID and the trueness bias are due to rounding

All data were acquired with two different resolution settings of the Q Exactive HF orbitrap mass spectrometer, i.e., 30 and 120 K, because of previous reports of spectral accuracy depending upon resolution [16, 17]. In case of selenomethionine, neither precision nor trueness bias of the ID determination was influenced by the resolution (Table 1). However, we observed a consistent trueness bias, i.e., underestimation of low abundance isotopologues together with an overestimation of the highest abundance isotopologues for the higher mass range; as already described by Su et al. [16], it was more pronounced at higher resolution. In fact, when analyzing NAD^+^ and ATP (see ESM, Table S2 and Fig. S2) of natural isotopic abundance, the analogous trend was observed. Again, an overestimation of the highest abundance isotopologue, which was more pronounced at higher resolution, was found. The overestimation of the M0 at 120 K was 3.7% and 4.6% for NAD^+^ and ATP, respectively. One contribution to this bias could be the background correction during raw file generation (generation from mass spectra from transients). In that case, higher numbers of low abundance isotopologues (occurring with increased resolution) lead to a more pronounced effect on the trueness bias. When analyzing isotopically enriched NAD^+^, excellent CID accuracies at both resolution settings were observed (see Table 2 and ESM Fig. S2). Further studies on spectral accuracy of organic molecules are required to understand and prevent the processes leading to overestimation of M0 isotopologues.

**Table 2:** Predicted CIDs (CID_pred_) and measured CIDs (CID_meas_) with trueness bias and precision (*N* = 4) of NAD^+^ isotopologues in the undiluted labeled in-house reference material measured via reversed-phase chromatography (HSS T3 column) in positive ESI mode at two resolutions in comparison to the predicted CIDs

Isotopologue	CID_pred_	120 K			30 K		
		CID_meas_	Trueness bias	Precision	CID_meas_	Trueness bias	Precision
NAD^+^_M0	0.0	0.0	0.0	0.00	0.0	0.0	0.00
NAD^+^_M1	0.0	0.0	0.0	0.00	0.0	0.0	0.00
NAD^+^_M2	0.0	0.0	0.0	0.00	0.0	0.0	0.00
NAD^+^_M3	0.1	0.0	−0.1	0.00	0.0	−0.1	0.00
NAD^+^_M4	0.3	0.2	−0.1	0.04	0.2	−0.1	0.02
NAD^+^_M5	1.0	1.0	0.0	0.04	0.9	−0.2	0.02
NAD^+^_M6	2.7	2.6	−0.1	0.04	2.5	−0.2	0.08
NAD^+^_M7	5.7	5.6	−0.2	0.13	5.5	−0.2	0.04
NAD^+^_M8	9.9	9.9	0.0	0.15	9.7	−0.3	0.04
NAD^+^_M9	14.2	14.1	−0.1	0.12	13.9	−0.4	0.10
NAD^+^_M10	16.9	16.9	0.0	0.20	16.6	−0.3	0.04
NAD^+^_M11	16.7	16.7	−0.1	0.02	16.8	0.0	0.10
NAD^+^_M12	13.8	13.9	0.1	0.11	14.1	0.3	0.09
NAD^+^_M13	9.5	9.6	0.1	0.06	10.0	0.5	0.09
NAD^+^_M14	5.4	5.6	0.2	0.14	5.8	0.5	0.04
NAD^+^_M15	2.5	2.6	0.1	0.08	2.8	0.3	0.03
NAD^+^_M16	0.9	1.0	0.1	0.06	1.0	0.1	0.03
NAD^+^_M17	0.3	0.3	0.0	0.01	0.2	0.0	0.04
NAD^+^_M18	0.1	0.0	0.0	0.02	0.0	−0.1	0.00
NAD^+^_M19	0.0	0.0	0.0	0.00	0.0	0.0	0.00
NAD^+^_M20	0.0	0.0	0.0	0.00	0.0	0.0	0.00
NAD^+^_M21	0.0	0.0	0.0	0.00	0.0	0.0	0.00

All values are given in percent (%)

### Using a ^13^C-labeled in-house reference material for evaluation of different LC-MS methods

In the next step, method validation considered the actual target metabolites since trueness and precision of measured CIDs are not only instrument-dependent but also compound- and isotopologue-dependent. Following the ideas of Portais and co-workers [13, 19], a ^13^C-labeled in-house reference material produced in yeast was introduced. *P. pastoris* was grown in a defined ^12^C/^13^C-labeled medium. Thereby, the relative abundance of all isotopologues of a certain metabolite extracted from the yeast cells followed a binomial distribution. CIDs were predicted with the actual content of ^13^C determined by NMR (49.755% ± 0.009%) before fermentation. The predicted carbon isotopologue abundance was associated with an uncertainty which was obtained considering the uncertainty of the NMR measurement. This uncertainty was propagated for all compounds with different numbers of carbon atoms for each isotopologue. The calculation was based on the model equation described elsewhere [13] and error propagation by Monte Carlo simulation. Experimental CIDs were obtained by a targeted evaluation workflow via chromatographic peak areas. These data were corrected for natural isotope abundance by the R package AccuCor [16] excluding correction for carbon as mentioned above.

The in-house reference material was characterized for primary metabolites using three different approaches. More specifically, the chromatographic separations involved a fully wettable RP column (HSS T3), a zwitterionic HILIC column (ZIC pHILIC), and anion-exchange chromatography (AS11-HC).

Table S3 (see ESM) gives an overview on the metabolite panel investigated in the yeast-derived in-house reference material, retention times, and the preferred polarity with respect to the specific separation. Overall, for CID determination negative mode was beneficial in the case of organic acids, phosphorylated carbohydrates, and nucleotides. For amino acids, positive ionization yielded higher intensities on average.

Most of the metabolites commonly investigated in tracer studies [2, 9–11] could be found in the ^13^C-labeled in-house reference material produced in yeast with at least one of the applied methods (Table S3). Detailed information showing CID accuracies and precisions for each metabolite is given in the ESM (ESM2.xlsx). As can be readily observed, agreement with the theoretically predicted CIDs was excellent overall. For the pattern present in the in vivo synthesized materials, the CIDs were determined with typical precisions between 0.02% and 1% and a low trueness bias of less than 1%. As an example, the four most important metabolic readouts for TCA cycle studies are shown in Table 3 and ESM Fig. S3. CIDs for alpha-ketoglutarate (AKG) and malate (Mal) were in perfect agreement with the predicted CIDs (trueness bias less than 1%) and excellent precisions of less than 0.5% (*N* = 4) were obtained. For citrate (Cit) a small contamination on the M0 was observed whereas succinate (Suc) showed a high abundance contamination by the unlabeled compound, which is in agreement with previously reported data for an extract of *P. augusta* [13]. As a result of their much higher ionization efficiency in negative mode, CIDs in positive mode were not calculated.

**Table 3:** Predicted CIDs with predicted error from NMR measurements and measured CIDs with precision (*N* = 4; for IC, *N* = 3) of alpha-ketoglutarate (AKG), citrate (Cit), malate (Mal), and succinate (Suc) in the undiluted labeled in-house reference material measured via reversed-phase chromatography (HSS T3), HILIC, and IC in negative ESI mode at 120 K resolution

		Predicted		HSS T3		pHILIC		IC	
		CID	Error	CID	Precision	CID	Precision	CID	Precision
AKG	M0	3.202	0.0028	3.61	0.09	3.64	0.04	3.37	0.13
	M1	15.855	0.0084	15.89	0.23	16.12	0.09	15.32	0.04
	M2	31.401	0.0054	31.85	0.31	31.46	0.15	32.36	0.11
	M3	31.096	0.0057	31.15	0.50	30.94	0.15	31.86	0.11
	M4	15.396	0.0083	14.83	0.12	15.07	0.15	14.67	0.09
	M5	3.049	0.0027	2.68	0.09	2.77	0.04	2.42	0.03
Cit	M0	1.609	0.0017	2.85	0.04	ND^a^	ND^a^	2.73	0.01
	M1	9.560	0.0067	9.38	0.12	10.78	0.13	9.38	0.02
	M2	23.666	0.0083	23.63	0.09	23.80	0.26	23.48	0.03
	M3	31.248	0.0002	31.06	0.02	32.32	0.15	31.28	0.02
	M4	23.208	0.0084	23.14	0.21	23.46	0.08	23.27	0.02
	M5	9.193	0.0066	8.79	0.05	8.57	0.05	8.90	0.02
	M6	1.517	0.0016	1.15	0.02	1.06	0.18	0.97	0.02
Mal	M0	6.373	0.0045	7.50	0.11	7.74	0.18	7.37	0.08
	M1	25.245	0.0089	24.41	0.14	24.65	0.32	24.72	0.02
	M2	37.498	0.0001	37.41	0.15	37.14	0.15	37.05	0.03
	M3	24.755	0.0089	24.57	0.14	24.62	0.11	24.78	0.09
	M4	6.129	0.0044	6.12	0.06	5.85	0.10	6.08	0.03
Suc	M0	6.373	0.0045	28.94*	1.02	31.87*	0.86	29.94*	0.51
	M1	25.245	0.0089	19.11	1.20	18.51	0.18	19.28	0.16
	M2	37.498	0.0001	26.84	0.63	26.73	0.52	27.84	0.27
	M3	24.755	0.0089	18.63	0.61	17.24	0.95	17.69	0.04
	M4	6.129	0.0044	6.47	0.12	5.65	0.34	5.25	0.11

*Succinate measurements indicate the presence of an unlabeled contaminant. All values are given in percent (%)

^a^ND indicates that the peak could not be integrated because of an interfering peak

### Employing extraction blanks and an unlabeled sample to identify contaminants and interferences

Most metabolites present in the in-house reference material produced in yeast were in good agreement with the predicted carbon isotopologue distributions. However, some metabolites, such as succinate, showed a significant deviation from the predicted pattern. Hence, procedural blanks and the sample matrix of a biologically relevant sample (mammalian cancer cell extract) were investigated to rule out exogenous metabolite sources. The study confirmed that significant amounts of lactate, malate, pyruvate, succinate, citrate, and 2-hydroxyglutarate (2HG) were introduced by sample preparation (extraction blanks from 6-well plates, see ESM Table S4). The highest amounts were found for succinate and 2HG. Only lactate was also detected upon testing the solvents used for extraction. Washing materials involved in sample preparation, such as culture plates, could reduce those contaminations but this was not further investigated in this study. Our findings are in agreement with previous studies reporting a contamination of bacteria and yeast extracts with unlabeled small organic acids [13]. Furthermore, some amino acids showed peak areas between 0.1% and 5% of the area in the HCT 116 extract (details for all investigated metabolites are shown in ESM Table S4). After correction for natural isotope abundance during data analysis, this contamination with naturally abundant metabolites will only affect the M0. Thus, by excluding the M0 from data analysis and only investigating (ratios of) M1 and heavier isotopologues, the results will not be influenced. However, as soon as M0 or pool size is taken into account, this contribution from blank samples has to be considered.

Finally, method validation in tracer studies should include an unlabeled sample to exclude interferences from other molecules present in the sample matrix, especially when the investigated model organism differs from the organism used for the production of the labeled reference material. In addition to potentially interfering sample components, the sample preparation protocols for yeast and mammalian cells differed. Thus, we evaluated all isotopologues of interest in the target list in ESM Table S3 regarding potential interferences considering the exemplary HCT 116 human colon carcinoma cell extract. When considering a retention time window of ±0.5 min and a mass accuracy of 5 ppm, three metabolites were affected: an interference with the M3 isotopologue of 2HG accounted for 10% of the overall CID with the reversed-phase separation in negative mode (RT difference 0.3 min). In HILIC separation, the M4 of methionine showed an interference which accounted for 0.2% and 2% in negative and positive mode, respectively (RT difference 0.4 min). Lastly, interferences with the M3 and M4 of tyrosine constituted 2% and 0.1% of the CID with RP separation in positive mode. These data further show the importance of chromatographic separation and narrow retention time windows in data analysis.

### Choice of separation method

In this study, we selected the target metabolites on the basis of a review of stable isotope tracer experiments [2]. The reversed-phase chromatography implementing the fully wettable stationary phase enabled retention of many crucial polar metabolites [2, 9–11] such as organic acids. Sugar phosphates and nucleotides eluted in the void volume or could not be detected at all. Despite the poor retention of many amino acids, CIDs in our in-house reference material were measured accurately. The approach based on zwitterionic HILIC separation at alkaline pH provided excellent retention and sensitivity for polar metabolites [27, 28]. Anion-exchange chromatography proved to be very suitable for the analysis of organic acids, sugar phosphates, and nucleotides but not for amino acids since most of them were lost in the suppressor as a result of their positive charge. Compared to previously published methods [27], a rather short gradient of 22 min was applied for the pHILIC separation. As a result, some critical isomers (such as 2PG/3PG and the hexose phosphates) were not separated as was the case for the two other chromatographic separations. Only G6P could be separated from F6P and G1P, which also coeluted, with ion chromatography. However, for most (qualitative) tracer studies, this isomer selectivity is not required. 2PG is directly downstream of 3PG in glycolysis; therefore, the labeling of those two metabolites will not differ if their conversion is not a rate-limiting step and they can thus be measured as a sum. Glucose could only be detected as a sum of hexoses in all investigated methods. Again, in most cases of qualitative tracer studies, the contribution of other hexoses to glucose is negligible, even in the case of studying the formation of glucose from labeled glutamine or lactate in gluconeogenesis [2]. The pentose phosphates ribose-5-phosphate, ribulose-5-phosphate, and xylose-5-phosphate were not separated. Despite this fact, the question whether the oxidative or the non-oxidative branch of the pentose phosphate pathway (PPP) is active could be addressed by measuring M1 or M2 of ribose-5-phosphate formed using [1,2-^13^C]glucose as substrate. For more sophisticated quantitative flux experiments, separation of isomers, especially of phosphorylated carbohydrates, is crucial. In this case alternative separations are available [27, 29, 30].

Overall, the three methods were shown to be similarly suitable for the investigated target metabolites in the ^13^C-labeled in-house reference material, since the largest error in CID determination is derived from contaminations introduced during sample preparation (see Fig. S3 and ESM Table S4). However, as can be inferred from the data shown in the ESM (ESM2.xlsx), differences regarding trueness and precision were observed for some metabolites. When taking a closer look at the data of, e.g., fructose-1,6-bisphosphate, one can easily see that the deviation of the pattern measured with the reversed-phase method is due to missing M0 and M6 peaks. This indicates the poor sensitivity for compounds eluting in the void volume. The same was observed for lysine. Many other amino acids, however, eluted in the void volume of the RP separation, but still gave excellent agreement with the predicted pattern (alanine, arginine, aspartate, glutamine, glycine, and proline in positive mode). These metabolites were very abundant (greater than 1 μM, see ESM Table S5) in our in-house reference material. This points to the general trend that signal intensity positively affects the accuracy of CID determination. High intensities can be achieved by high abundance metabolites and/or sensitive methods. Since the abundance in biological samples can hardly be influenced (preconcentration of samples might help but is inevitably linked to an increase of matrix), choosing sensitive methods and providing sufficient retention are essential. The concentration ranges of the investigated metabolites observed in the HCT 116 extract are given in ESM Table S5. Linear calibration ranges for the multi-metabolite standard mix were calculated for the different chromatographic separations (ESM Table S6). The lower value of these calibration ranges greatly differed between the LC method indicating which method provides the best sensitivity for a specific metabolite. This data also correlates with the intensities (peak height) obtained from replicate determinations of the 10 μM multi-metabolite standard shown in ESM Fig. S4. Since this standard mix did not account for matrix effects, we additionally compared the intensities obtained from the cancer cell extracts (mammalian cancer cells, 2.5 × 10^5^ seeded cells after 24 h in 200 μL). As can be seen in ESM Fig. S5, compounds in the void volume were strongly affected by suppression caused by the sample matrix leading to lower intensities. From this simplified metric, optimum methods for specific tracer experiments could be inferred.

## Conclusions

The application of standard materials showing CIDs representative of ^13^C tracer studies is crucial in order to assess the paramount analytical metrics of uncertainty and accuracy. We propose a novel validation scheme allowing for validation of instrumental performance for ideal isotopologue distributions applicable to diverse chromatographic separations and for validation of the accuracy of carbon isotopologue distributions in biological samples. Owing to its isotopic pattern, selenomethionine constituted an ideal compound to assess a mass spectrometer’s performance regarding the measurement of accurate and precise isotopologue distributions, while a ^13^C-labeled in-house reference material produced in *P. pastoris* proved to be an ideal material to assess CID accuracy as it contained 40 out of 45 metabolites commonly investigated in tracer studies. More specifically, by investigating the CIDs of these primary metabolites using the most commonly applied chromatographic separations (namely IC, HILIC, and RP) applied in tracer studies, we could point out the challenges arising from different factors such as method-related parameters, sample properties, and background introduced by sample preparation. Although experimental design of metabolite tracer studies should preferably lead to a high degree of ^13^C enrichment, isotopologue distributions with accuracies and precisions as assessed for metabolites of natural abundance have to be considered in cases of very low ^13^C enrichment.

## Electronic supplementary material

ESM 1 (PDF 726 kb)

ESM 2 (XLSX 326 kb)

## Data Availability

The datasets generated during the current study are available from the corresponding author on reasonable request.
