# Supplementary material for: Proposing a validation scheme for 13C metabolite tracer studies in high-resolution mass spectrometry
Source: Anal Bioanal Chem. 2019 Apr 10;411(14):3103–13. doi: 10.1007/s00216-019-01773-7 (PMC6526147; doi:10.1007/s00216-019-01773-7)
Supplement: Supplementary file 1 — (PDF 726 kb) [file 216_2019_1773_MOESM1_ESM.pdf]

Analytical and Bioanalytical Chemistry

Electronic Supplementary Material

Proposing a validation scheme for ^{13}C metabolite tracer studies in high-resolution mass spectrometry

Michaela Schwaiger-Haber, Gerrit Hermann, Yasin El Abiead, Evelyn Rampler,
Stefanie Wernisch, Kelli Sas, Subramaniam Pennathur, Gunda Koellensperger

Additional file available under 10.1007/s00216-019-01773-7

Table S1 ESI parameters of Q Exactive HF for the three different LC methods

Parameter	RP<br>*HSS T3*	HILIC<br>*ZIC pHILIC*	IC<br>*AS11-HC*
**Sheath gas**	48	40	50
**Auxiliary gas**	11	3	14
**Spray voltage [kV] +/-**	3.5 / 2.8	3.5 / 2.8	2.8
**Capillary temperature [°C]**	255	280	270
**Auxiliary gas heater [°C]**	410	320	380
**S-Lens RF level**	30	30	45

Table S2 Measured CIDs (CID_{meas}) with CID accuracy and CID precision (N=4) of NAD^+ isotopologues of unlabeled NAD^+ in a 10 µM multi-metabolite mix measured via reversed phase chromatography (HSS T3 column) in positive ESI mode and ATP measured via anion-exchange chromatography in negative ESI mode at two resolutions in comparison to the theoretical CIDs due to natural isotope abundance (CID_{theor}). All values are given in per cent (%)

		120 K			30K		
**Isotopologue**	**CID_{theor}**	**CID_{meas}**	**Trueness bias**	**Precision**	**CID_{meas}**	**Trueness bias**	**Precision**
**NAD^+_M0**	**75.0**	78.7	3.7	0.11	77.8	2.9	0.24
**NAD^+_M1**	**19.6**	17.6	-2.0	0.13	19.1	-0.5	0.34
**NAD^+_M2**	**4.6**	3.5	-1.1	0.11	3.2	-1.5	0.22
**NAD^+_M3**	**0.8**	0.2	-0.6	0.05	0.0	-0.8	0.00
**ATP_M0**	**85.6**	90.2	4.6	0.23	87.2	1.6	0.26
**ATP_M1**	**11.4**	8.2	-3.2	0.02	10.9	-0.5	0.07
**ATP_M2**	**3.0**	1.6	-1.4	0.24	1.9	-1.1	0.28

Table S3 List of metabolites recovered in the yeast-based in-house reference material. The list includes retention times on the three different chromatographic separations and preferred ionization mode for measuring accurate CIDs in the labeled reference material (n.d. in the polarity column indicates that the compound was not detected in the reference material). Values in brackets represent second choice which are possible but not ideal (see CIDs in the electronic supplementary material, ESM2.xlsx) and should only be considered if no other method/polarity is possible

		RP		HILIC		IC		
**Metabolite**	**Abbr.**	**RT [min]**	**Pola-rity**	**RT [min]**	**Pola-rity**	**RT [min]**	**Pola-rity**	**Comment**
**Organic acids**								
2-Hydroxyglutaric acid	2HG	2.8	n.d.	4.2	-	6.3	-	
alpha-Ketoglutarate	AKG	2.3	-	4.3	-	8.6	-	
Citrate	Cit	3.2	-	5.8	-	11.5	-	pHILIC neg: M0 background
Fumarate	Fum	3.7	-	2.1	-	9.2	-	
Lactate	Lac	2.4	-	1.7	-	2.5	-	M0 contaminant
Malate	Mal	1.9	-	4.6	-	6.8	-	
Pyruvate	Pyr	1.8	(-)	1.5	-	3	-	
Succinate	Suc	4.2	-	4.2	-	6.7	-	M0 contaminant
**(Phosphorylated) carbohydrates**								
2/3-Phosphoglycerate	3PG	1.4	-	5.4	-	11	-	
Dihydroxyacetone-phosphate	DHAP	1.3	n.d.	4.2	-	n.d.		
Fructose-1,6-bisphosphate	FBP	1.4	(-)	6.2	-	13.6	-	
Hexose-6-phosphate	Hex6P	1.3	n.d.	5	-	8.6	-	
Hexose	Hexose	1.2	(-)	3.5	-	2.2	-	
Pentose-5-phosphate	Pen5P	1.3	n.d.	4.4	-	9.2	-	
**Amino acids**								
Alanine	Ala	1.2	+	3.7	+/-	n.d.		
Arginine	Arg	1.1	+	9	+/-	n.d.		
Asparagine	Asn	1.1	+	4	-	n.d.		pHILIC: 120 K required to avoid interference
Aspartate	Asp	1.2	+	3.9	+/-	6	-	
Cysteine	Cys	1.3	n.d.	n.d		n.d.		
Glutamine	Gln	1.2	+	3.9	+/-	n.d.		
Glutamate	Glu	1.2	+	3.7	+/-	n.d	n.d.	
Glycine	Gly	1.1	+	4.2	+	n.d.		
Histidine	His	1.1	+	7	+	n.d.		pHILIC: extremely broad peak
Isoleucine	Ile	4.3	+/(-)	2.3	+/-	n.d.		pHILIC: Leu+Ile
Leucine	Leu	4.5	+/(-)	2.3	+/-	n.d.		pHILIC: Leu+Ile
Lysine	Lys	1	(+)	8.5	+/-	n.d.		
Methionine	Met	2.5	+/(-)	2.5	+/((-))	n.d.		
Phenylalanine	Phe	5.7	+/(-)	2	+/(-)	n.d.		
Proline	Pro	1.4	+	2.9	+/(-)	n.d.		
Serine	Ser	1.1	+	4.2	+/((-))	n.d.		pHILIC: 120 K required

Metabolite	Abbr.	RP		HILIC		IC		Comment
		RT [min]	Pola-rity	RT [min]	Pola-rity	RT [min]	Pola-rity	
Threonine	Thr	1.2	(+)	3.5	+/-	n.d.		
Tryptophan	Trp	6.3	+/(-)	2.6	-	n.d.		
Tyrosine	Tyr	4.5	+/(-)	3.1	+	n.d.		
Valine	Val	2	+	2.8	-	n.d.		
**Nucleotides**								
Adenosine triphosphate	ATP	n.d.		5.2	-	15.7	-	
Guanosine triphosphate	GTP	n.d.		6.5	-	17.6	-	
Thymidine triphosphate	TTP	n.d.	n.d.	4.9	n.d.	16.4	-	
Uridine triphosphate	UTP	n.d.	n.d.	5.8	(-)	16.7	-	
**Others**								
Glutathione, reduced	GSH	2.8	+	3.5	n.d.	10.9	-	
N-Acetylserine	NAS	2	n.d.	2.1	n.d.	2.3	n.d.	
N-Acetylaspartate	NAA	2.7	n.d.	3.8	n.d.	5	n.d.	Other metabolite in in-house ref.mat.
NAD^+	NAD+	4.6	+	3.4	n.a.	n.d.		NAD^+/NADH not well separated on pHILIC
NADH	NADH	5.2	+/-	3.3	n.a.	n.d.		NAD^+/NADH not well separated on pHILIC
Selenomethionine	Se-Met	3.4	n.d.	2.5	n.d.	n.d.		

Table S4 Occurrence of the investigated metabolites in their natural abundant form in extraction blanks (extraction of a 6-well plate without any cells/media) detected by the different chromatographic separations. The values are given as the ratio of the peak area in the extraction blank divided by the peak area in the HCT 116 extract in per cent [%] to show the potential contribution of contaminants to the carbon isotopologue distribution of a certain metabolite

Metabolite	Abbr.	RP pos	RP neg	pHILIC pos	pHILIC neg	IC neg
**Organic acids**						
2-Hydroxyglutaric acid	2HG	n.a.	*12*	n.a.	*43**	*17**
alpha-Ketoglutarate	AKG	n.a.	-	n.a.	-	*0.5*
Citrate	Cit	n.a.	*0.7*	n.a.	-	*0.4*
Fumarate	Fum	n.a.	*0.8*	n.a.	*6*	-
Lactate	Lac	n.a.	*2*	n.a.	*4*	*1.2*
Malate	Mal	n.a.	*4*	n.a.	*9*	*0.7*
Pyruvate	Pyr	n.a.	*2.9*	n.a.	-	*12**
Succinate	Suc	n.a.	*25*	n.a.	*30*	*7*
**(Phosphorylated) carbohydrates**						
2/3-Phosphoglycerate	3PG	n.a.	-	n.a.	-	*0.1*
Dihydroxyacetone-phosphate	DHAP	n.a.	-	n.a.	-	-
Fructose-1,6-bisphosphate	FBP	n.a.	-	n.a.	-	-
Hexose-6-phosphate	Hex6P	n.a.	-	n.a.	-	-
Hexose	Hexose	n.a.	*17*	n.a.	-	*0.1*
Pentose-5-phosphate	Pen5P	n.a.	-	n.a.	-	-
**Amino acids**						
Alanine	Ala	*2*	-	*1.1*	-	n.a.
Arginine	Arg	*3*	-	-	-	n.a.
Asparagine	Asn	*0.5*	-	-	-	n.a.
Aspartate	Asp	*3*	*1*	-	-	*0.2*
Cysteine	Cys	n.a.	-	n.a.	-	n.a.
Glutamine	Gln	*0.2*	*0.1*	-	-	n.a.
Glutamate	Glu	*0.3*	*0.1*	-	-	*2*
Glycine	Gly	-	-	*0.5*	-	n.a.
Histidine	His	*2*	-	-	-	n.a.
Isoleucine	Ile	0.1	-	-	-	n.a.
Leucine	Leu	0.1	-	-	-	n.a.
Lysine	Lys	*1*	-	-	-	n.a.
Methionine	Met	-	-	-	-	n.a.
Phenylalanine	Phe	0.1	-	*1*	-	n.a.
Proline	Pro	0.1	-	*0.1*	-	n.a.
Serine	Ser	*5*	*4*	*1*	*3*	n.a.
Threonine	Thr	*4*	*1*	*1*	*1*	n.a.

Metabolite	Abbr.	RP pos	RP neg	pHILIC pos	pHILIC neg	IC neg
Tryptophan	Trp	-	-	-	-	n.a.
Tyrosine	Tyr	*0.1*	-	*0.3*	-	n.a.
Valine	Val	-	-	-	-	n.a.
**Nucleotides**						
Adenosine triphosphate	ATP	n.a.	n.a.	n.a.	-	-
Guanosine triphosphate	GTP	n.a.	n.a.	n.a.	-	-
Thymidine triphosphate	TTP	n.a.	n.a.	n.a.	-	-
Uridine triphosphate	UTP	n.a.	n.a.	n.a.	-	-
**Others**						
Glutathione, reduced	GSH	-	-	-	-	-
N-Acetylserine	NAS	-	*2**	n.a.	n.a.	*2*
N-Acetylaspartate	NAA	-	-	n.a.	n.a.	-
NAD^+	NAD+	-	-	n.a.	n.a.	n.a.
NADH	NADH	-	-	n.a.	n.a.	n.a.
Selenomethionine	Se-Met	n.a.	n.a.	n.a.	n.a.	n.a.

* Peak eluted not at exactly same retention time but within ± 0.5 min

Table S5 Concentration ranges of the investigated metabolites in the HCT 116 cell extract (2.5 x 10^5 seeded cells after 24 h in 200 µL) and in our in-house reference material (for the highest abundant isotopologue M_{max})

Metabolite	Abbr.	HCT 116 concentration range* [µM]	In-house reference material concentration range* of M_{max} [µM]
**Organic acids**			
2-Hydroxyglutaric acid	2HG	0.1 - 1	0.5 - 5
alpha-Ketoglutarate	AKG	0.1 - 1	1 - 10
Citrate	Cit	1 - 10	1 - 10
Fumarate	Fum	0.1 - 1	0.5 - 5
Lactate	Lac	10 - 100	0.1 - 0.5
Malate	Mal	1 - 10	1 - 10
Pyruvate	Pyr	0.5 - 5	0.1 - 1
Succinate	Suc	0.5 - 5	0.5 - 5
**(Phosphorylated) carbohydrates**			
2/3-Phosphoglycerate	3PG	1 - 10	5 - 30
Dihydroxyacetone-phosphate	DHAP	1 - 10	0.1 - 1
Fructose-1,6-bisphosphate	FBP	1 - 10	1 - 10

Metabolite	Abbr.	HCT 116 concentration range* [µM]	In-house reference material concentration range* of M_{max} [µM]
Hexose-6-phosphate	Hex6P	1 - 10	0.1 - 0.5
Hexose	Hexose	50 - 150	1 - 10
Pentose-5-phosphate	Pen5P	0.5 - 5	0.1 - 1
**Amino acids**			
Alanine	Ala	1 - 10	5 - 50
Arginine	Arg	1 - 10	2 - 20
Asparagine	Asn	1 - 10	1 - 10
Aspartate	Asp	1 - 10	100 - 200
Cysteine	Cys	n.d.	n.d.
Glutamine	Gln	10 - 100	10 - 100
Glutamate	Glu	10 - 100	10 - 100
Glycine	Gly	5 - 15	10 - 100
Histidine	His	0.5 - 1.5	0.1 - 1
Isoleucine	Ile	1 - 10	0.5 - 5
Leucine	Leu	1 - 10	1 - 10
Lysine	Lys	1 - 10	1 - 10
Methionine	Met	1 - 10	0.1 - 0.5
Phenylalanine	Phe	1 - 10	0.1 - 1
Proline	Pro	1 - 10	1 - 10
Serine	Ser	10 - 100	0.1 - 1
Threonine	Thr	0.5 - 5	0.5 - 5
Tryptophan	Trp	0.1 - 1	0.05 - 0.5
Tyrosine	Tyr	1 - 10	0.1 - 1
Valine	Val	1 - 10	5 - 10
**Nucleotides**			
Adenosine triphosphate	ATP	1 - 10	10 - 50
Guanosine triphosphate	GTP	1 - 10	1 - 10
Thymidine triphosphate	TTP	0.1 - 1	0.1 - 0.5
Uridine triphosphate	UTP	1 - 10	1 - 10
**Others**			
Glutathione, reduced	GSH	10 - 100	0.5 - 5
N-Acetylserine	NAS	n.d.	n.d.
N-Acetylaspartate	NAA	n.d.	n.d.
NAD^+	NAD+	1 - 10	1 - 10
NADH	NADH	n.d.	1 - 10
Selenomethionine	Se-Met	n.d.	n.d.

* Absolute quantification was not possible as no internal standards were used for this study

Table S6 Linear calibration ranges and the correlation coefficients R^2 for the different chromatographic separations. All concentrations are given in µM for the of the metabolites present in the standard mix. R^2 values are based on external linear calibration functions

Metabolite	Abbr.	RP pos Cal range	RP pos R^2	RP neg Cal range	RP neg R^2	pHILIC pos Cal range	pHILIC pos R^2	pHILIC neg Cal range	pHILIC neg R^2	IC neg Cal range	IC neg R^2
**Organic acids**											
2-Hydroxyglutaric acid	2HG	n.a.	n.a.	0.01 - 25	1.000	n.a.	n.a.	0.01 - 5	0.995	0.01 - 10	0.995
alpha-Ketoglutarate	AKG	n.a.	n.a.	0.01 - 25	1.000	n.a.	n.a.	0.01 - 10	0.993	0.01 - 10	0.997
Citrate	Cit	n.a.	n.a.	0.01 - 5	0.995	n.a.	n.a.	0.1 - 10	0.994	0.01 - 10	0.995
Fumarate	Fum	n.a.	n.a.	0.01 - 25	0.998	n.a.	n.a.	0.01 - 10	1.000	0.1 - 10	0.996
Lactate	Lac	n.a.	n.a.	0.01 - 25	0.973	n.a.	n.a.	0.01 - 10	0.999	0.01 - 25	0.981
Malate	Mal	n.a.	n.a.	0.01 - 25	1.000	n.a.	n.a.	0.01 - 5	0.997	0.01 - 10	0.998
Pyruvate	Pyr	n.a.	n.a.	0.01 - 25	0.997	n.a.	n.a.	0.1 - 10	1.000	0.01 - 10	0.996
Succinate	Suc	n.a.	n.a.	0.01 - 25	0.999	n.a.	n.a.	0.01 - 10	1.000	0.01 - 10	0.996
**(Phosphorylated) carbohydrates**											
2/3-Phosphoglycerate	3PG	n.a.	n.a.	0.1 - 25	0.999	n.a.	n.a.	0.01 - 10	0.999	0.01 - 10	0.998
Dihydroxyacetone-phosphate	DHAP	n.a.	n.a.	0.01 - 5	0.996	n.a.	n.a.	0.01 - 10	1.000	0.01 - 10	0.997
Fructose-1,6-bisphosphate	FBP	n.a.	n.a.	5 - 25	0.988	n.a.	n.a.	0.1 - 10	0.994	0.01 - 10	0.997
Hexose-6-phosphate	Hex6P	n.a.	n.a.	0.01 - 5	0.994	n.a.	n.a.	0.01 - 10	1.000	0.1 - 10	0.996
Hexose	Hexose	n.a.	n.a.	0.01 - 1	0.953	n.a.	n.a.	0.1 - 10	0.986	0.01 - 25	0.998
Pentose-5-phosphate	Pen5P	n.a.	n.a.	0.01 - 5	0.995	n.a.	n.a.	0.01 - 10	1.000	0.1 - 10	0.986
**Amino acids**											
Alanine	Ala	0.01 - 10	0.990	0.1 - 10	0.999	0.01 - 5	0.992	0.1 - 10	0.997	n.a.	n.a.
Arginine	Arg	0.01 - 5	0.995	0.01 - 1	0.990	0.01 - 5	0.998	0.01 - 10	0.999	n.a.	n.a.
Asparagine	Asn	0.01 - 5	0.999	0.01 - 10	0.993	0.01 - 10	1.000	0.01 - 1	0.999	n.a.	n.a.
Aspartate	Asp	0.01 - 5	0.998	0.01 - 10	1.000	0.01 - 10	1.000	0.01 - 1	0.994	0.01 - 10	0.997
Cysteine	Cys	1 - 25	0.999	n.a.	n.a.	n.a.	n.a.	1 - 10	0.999	n.a.	n.a.
Glutamine	Gln	0.01 - 5	0.994	0.01 - 10	0.993	0.01 - 10	0.994	0.01 - 1	0.998	n.a.	n.a.

Metabolite	Abbr.	RP pos		RP neg		pHILIC pos		pHILIC neg		IC neg	
		Cal range	R^2	Cal range	R^2	Cal range	R^2	Cal range	R^2	Cal range	R^2
Glutamate	Glu	0.01 - 5	0.997	0.01 - 5	0.998	0.01 - 10	0.999	0.01 - 10	0.998	0.01 - 10	0.990
Glycine	Gly	0.1 - 5	0.995	0.1 - 5	0.992	0.1 - 5	0.998	0.1 - 10	0.999	n.a.	n.a.
Histidine	His	0.01 - 5	0.962	0.01 - 0.1	0.947	0.01 - 10	0.997	0.1 - 10	0.998	n.a.	n.a.
Isoleucine	Ile	0.01 - 25	0.996	0.1 - 10	0.995	0.01 - 10	0.999	0.01 - 10	1.000	n.a.	n.a.
Leucine	Leu	0.01 - 25	0.992	0.01 - 10	0.990	0.01 - 10	0.999	0.01 - 10	1.000	n.a.	n.a.
Lysine	Lys	0.01 - 5	0.988	0.1 - 5	0.994	0.1 - 5	0.998	0.1 - 5	1.000	n.a.	n.a.
Methionine	Met	0.01 - 25	1.000	0.01 - 10	0.992	0.01 - 10	0.993	0.01 - 10	0.998	n.a.	n.a.
Phenylalanine	Phe	0.01 - 25	0.997	0.01 - 5	0.999	0.01 - 10	0.999	0.01 - 10	1.000	n.a.	n.a.
Proline	Pro	0.01 - 10	0.993	5 - 25	0.999	0.01 - 5	0.991	0.1 - 10	0.999	n.a.	n.a.
Serine	Ser	0.01 - 10	0.985	0.01 - 10	0.992	n.a.	n.a.	0.01 - 0.1	0.998	n.a.	n.a.
Threonine	Thr	0.01 - 10	0.991	0.01 - 25	0.890	0.01 - 10	0.999	0.01 - 1	0.999	n.a.	n.a.
Tryptophan	Trp	0.01 - 25	0.999	0.01 - 25	0.990	0.01 - 10	1.000	0.01 - 10	1.000	n.a.	n.a.
Tyrosine	Tyr	0.01 - 25	0.992	0.01 - 25	0.895	0.01 - 10	0.993	0.01 - 10	0.999	n.a.	n.a.
Valine	Val	0.01 - 25	0.999	0.1 - 25	0.997	0.1 - 10	0.996	0.01 - 10	0.999	n.a.	n.a.
**Nucleotides**											
Adenosine triphosphate	ATP	n.a.	n.a.	n.a.	n.a.	n.a.	n.a.	0.1 - 10	0.998	0.1 - 10	0.999
Guanosine triphosphate	GTP	n.a.	n.a.	n.a.	n.a.	n.a.	n.a.	0.1 - 5	1.000	0.1 - 10	0.999
Thymidine triphosphate	TTP	n.a.	n.a.	n.a.	n.a.	n.a.	n.a.	0.1 - 10	0.998	0.01 - 10	0.998
Uridine triphosphate	UTP	n.a.	n.a.	n.a.	n.a.	n.a.	n.a.	0.01 - 10	0.996	0.1 - 10	1.000
**Others**											
Glutathione, reduced	GSH	0.01 - 25	0.994	0.01 - 10	0.994	0.01 - 10	1.000	0.01 - 10	1.000	0.01 - 10	0.991
N-Acetylserine	NAS	0.01 - 10	0.994	0.01 - 25	0.999	n.a.	n.a.	n.a.	n.a.	0.01 - 10	0.990
N-Acetylaspartate	NAA	0.01 - 10	0.992	0.01 - 25	1.000	n.a.	n.a.	n.a.	n.a.	0.01 - 10	0.997
NAD^+	NAD+	0.1 - 25	0.996	0.01 - 10	0.996	n.a.	n.a.	n.a.	n.a.	n.a.	n.a.
NADH	NADH	5 - 25	0.999	0.1 - 25	1.000	n.a.	n.a.	n.a.	n.a.	n.a.	n.a.
Selenomethionine	Se-Met	0.1 - 25	1.000	5 - 25	1.000	0.1 - 10	0.998	1 - 10	0.994	n.a.	n.a.

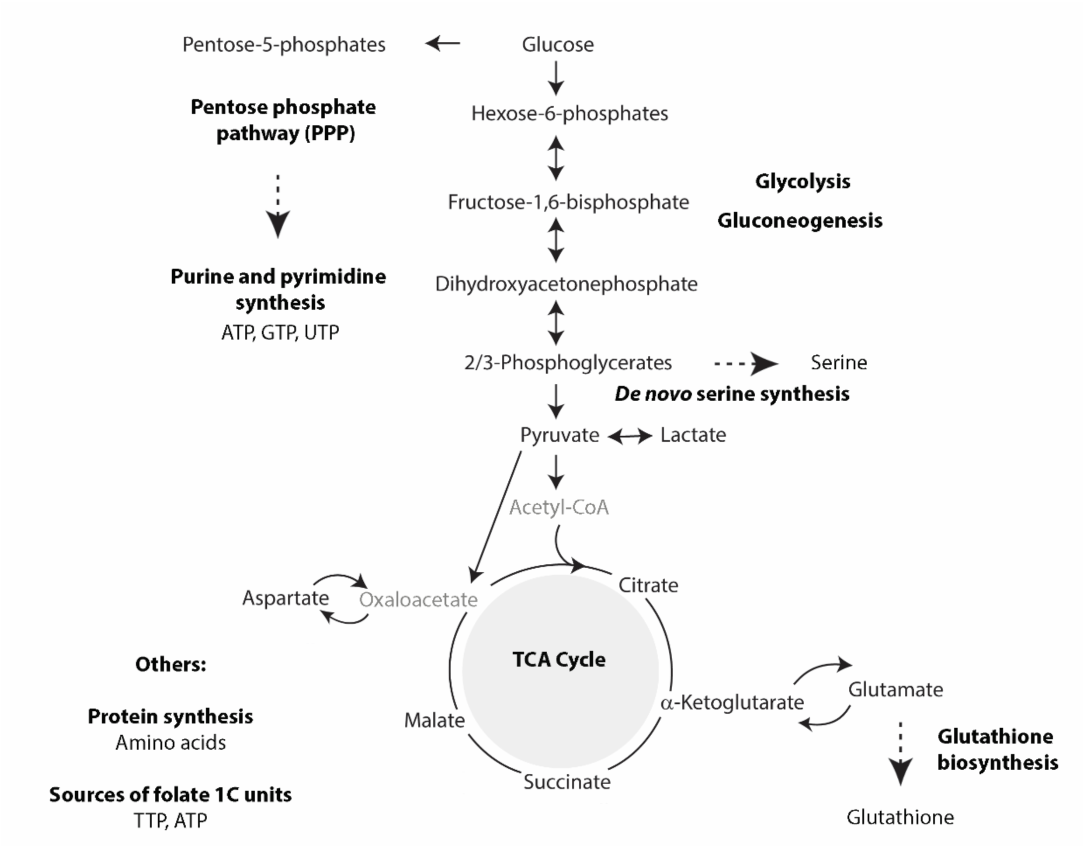

Fig. S1 Selected pathways considering the most commonly assessed metabolites in tracing experiments involving ^{13}C labeled substrates [2]. ATP adenosine triphosphate, GTP guanosine triphosphate, UTP uridine triphosphate, TTP thymidine triphosphate. Gray: not analyzed

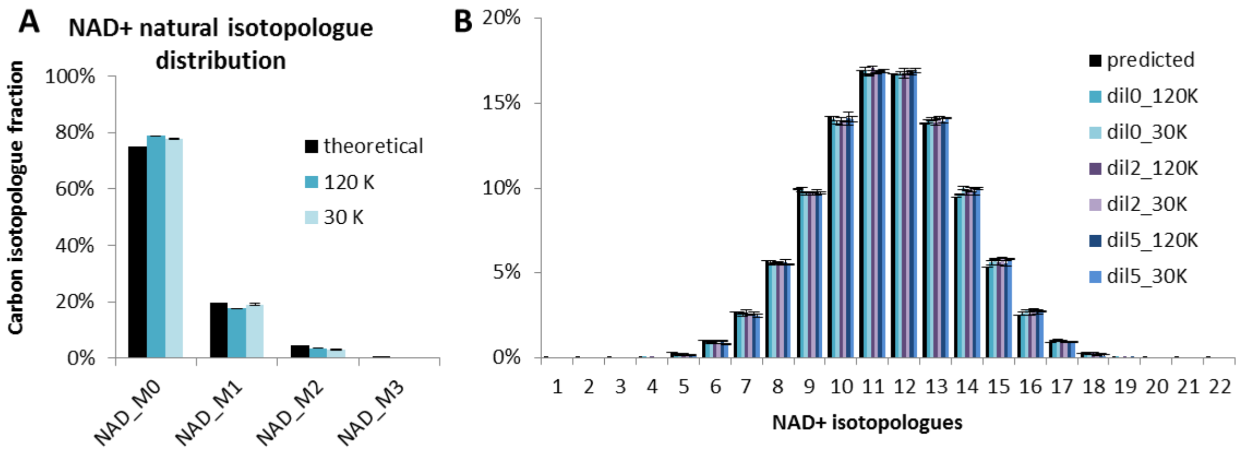

Fig. S2 Carbon isotopologue distribution (CID) accuracy of NAD+ acquired with two resolution settings (120 K and 30 K): A) Comparison of the measured CID of unlabeled NAD+ with the theoretical distribution (black) due to natural isotope abundance. B) Comparison of the measured CID in three different dilutions (dil0 - undiluted, dil2 - 1:2 diluted and dil5 - 1:5 diluted) of the labeled yeast extract with the predicted distribution (black). Error bars represent the standard deviation of the mean (N=4). Error bars for the in-house reference material were calculated by Monte Carlo simulation from the standard deviation of the NMR measurement of 12C/13C methanol used for the fermentation

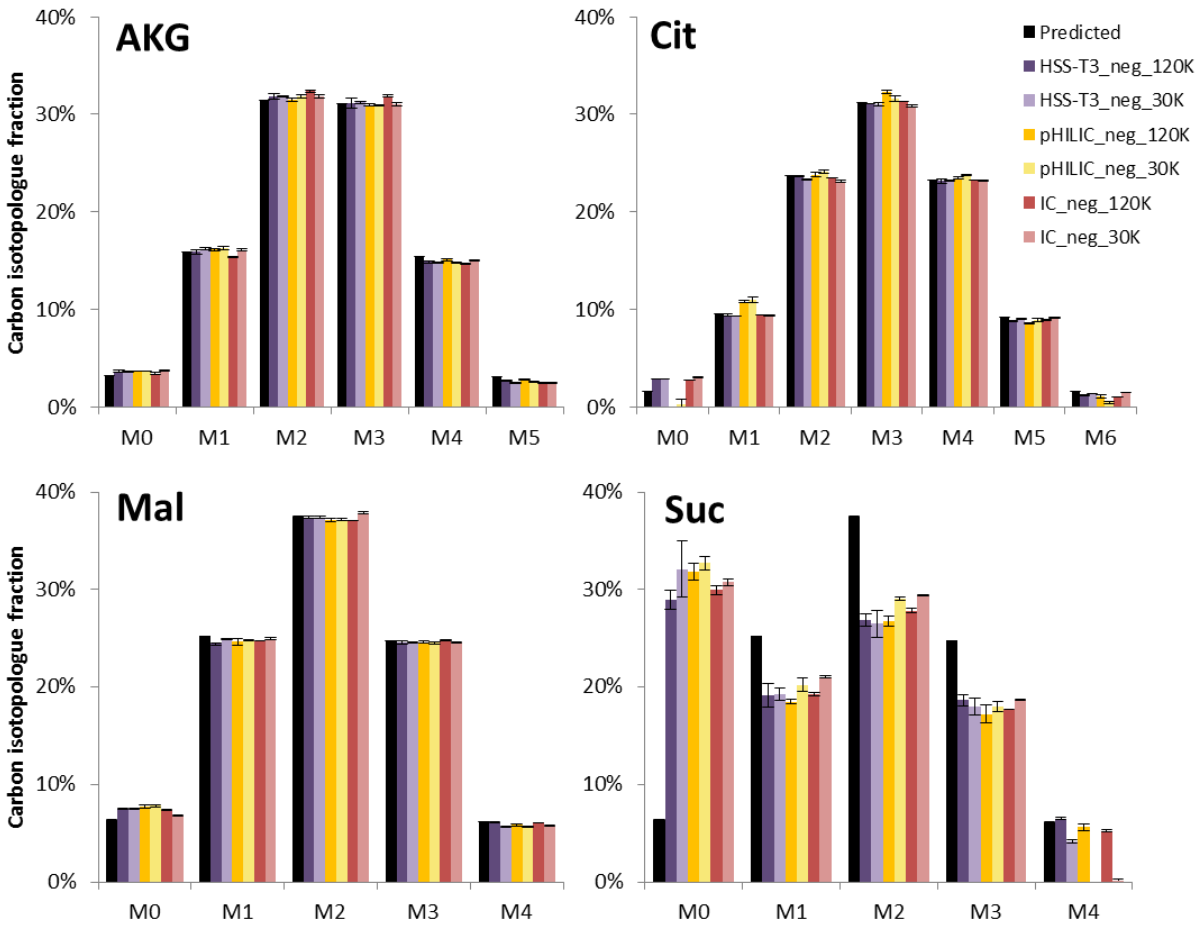

Fig. S3 CIDs of alpha-ketoglutarate (AKG), citrate (Cit), malate (Mal) and succinate (Suc) measured in the labeled in-house reference material with different LC separations in comparison to the predicted pattern. Succinate measurements indicate the presence of an unlabeled contaminant. Error bars represent the standard deviation of the mean (N=4, for IC: N=3)

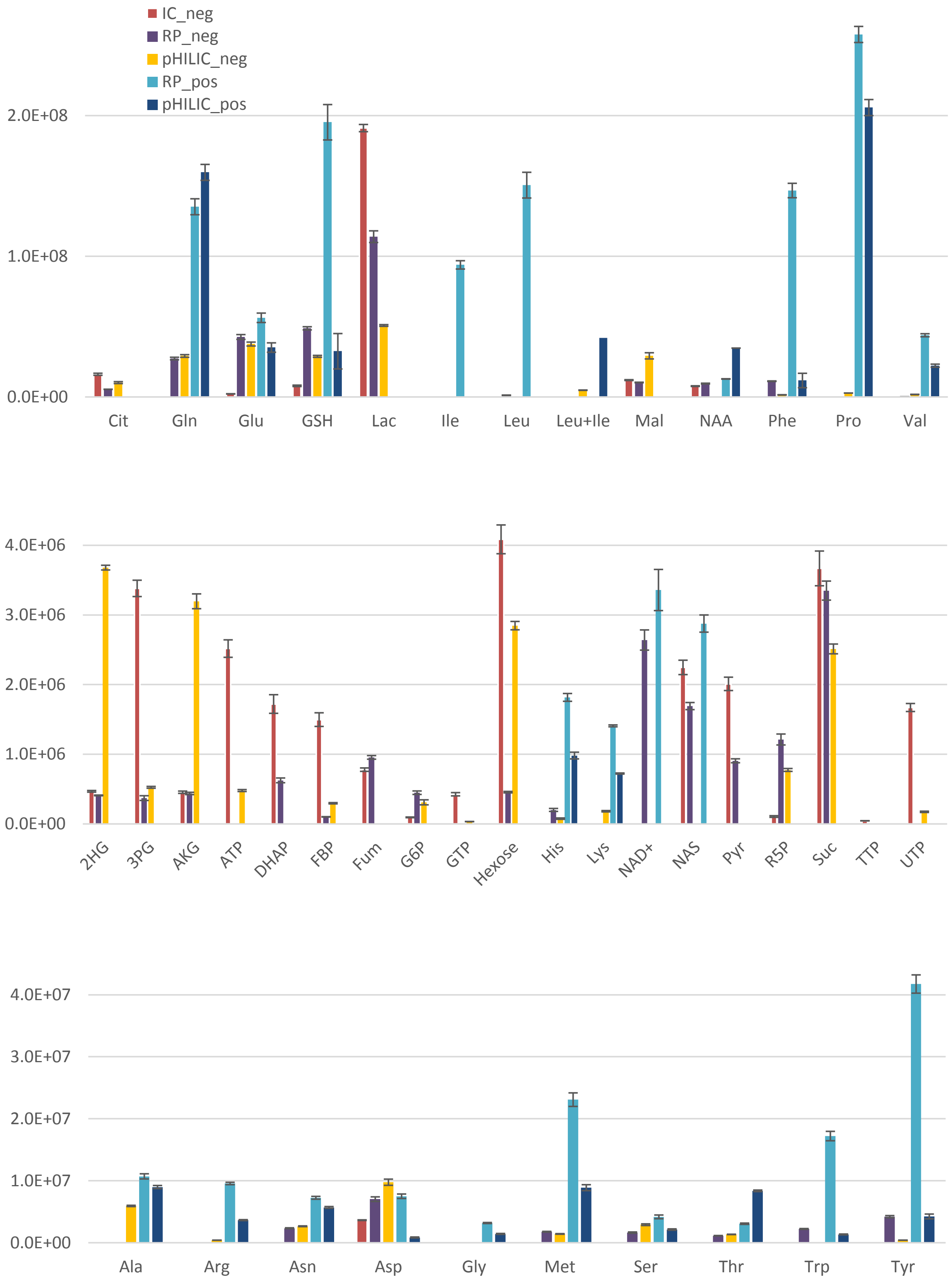

Fig. S4 Comparison of intensities measured in an extract of HCT 116 cells (2.5 x 10^5 seeded cells after 24 h extracted and reconstituted in 200 µL) from the three separations. Error bars denote the standard deviation of technical replicates (N=3). A list of abbreviations can be found in Table S3

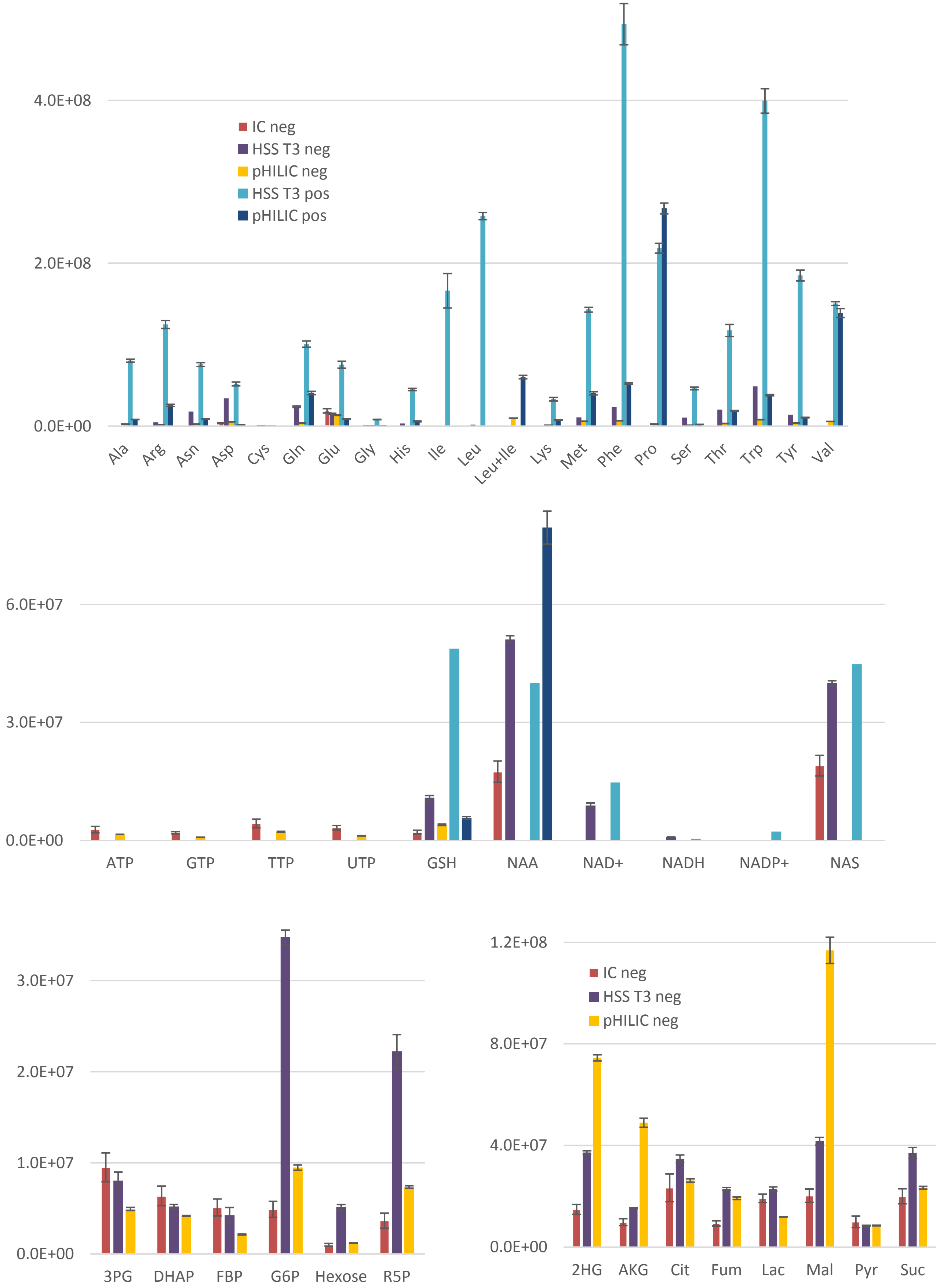

Fig. S5 Intensities of a 10 µM multi-metabolite mix measured with the three separations. Error bars denote the standard deviation of technical replicates (N=4). A list of abbreviations can be found in Table S3
